# Depression and Anxiety among Undergraduate Health Science Students: A Scoping Review of the Literature

**DOI:** 10.3390/bs13121002

**Published:** 2023-12-07

**Authors:** Gerald Agyapong-Opoku, Belinda Agyapong, Gloria Obuobi-Donkor, Ejemai Eboreime

**Affiliations:** 1 School of Health and Human Performance, Faculty of Health, Dalhousie University, Halifax, NS B3H 4R2, Canada; 2 Department of Psychiatry, University of Alberta, Edmonton, AB T6G 2B7, Canada; 3 Department of Psychiatry, Dalhousie University, Halifax, NS B3H 2E2, Canadaeboreime@ualberta.ca (E.E.)

**Keywords:** depression, anxiety, health sciences, students, prevalence

## Abstract

**Background:** Health science students in post-secondary institutions experience high levels of depression and anxiety due to increased stress levels, workload, low socioeconomic status, and history of family mental illness, among other factors. Given the significant negative impact that depression and anxiety can have on undergraduate health science students, it is essential to understand the prevalence and correlation of these conditions in this population. In light of this, this scoping review aims to identify, document, and analyze the literature on the prevalence and determinants of anxiety and depression among undergraduate health sciences students and identify gaps in knowledge for future research. **Methods:** This scoping review was planned and executed using the Preferred Reporting Items for Systematic Reviews and Meta-Analyses extension for the Scoping Reviews statement. A comprehensive and systematic search was carried out for five databases, namely MEDLINE, Scopus, EMBASE, CINAHL, and PubMed. **Results:** From the literature identified by our search strategy, the lowest prevalence for anxiety was 5.8%, and the highest was 82.6%, with a median of 44.25%. The prevalence of depression ranged from a high of 88.8% to a low of 2.1%, with a median value of 34.8%. Our analysis revealed that correlates of anxiety and depression among health science students include sociodemographic factors such as age, sex, gender, relationships, ethnicity, and family history, personal health conditions, and academic and socioeconomic issues. **Conclusions:** With the high incidence of anxiety and depression among health science students, there is an increasing need to find practical remedies to support these students. It is also essential for policymakers and university authorities to implement interventions such as supportive text messages and other strategies geared toward providing support and improving the psychological well-being of health science students.

## 1. Introduction

Anxiety and depression are globally common aspects of mental illness, affecting millions of individuals every year [1]. Depression (major depressive disorder or clinical depression) can be defined as a common, but serious, mood disorder associated with negative feelings which affects how you feel, the way you think, and how you act. Individuals suffering from depression experience persistent feelings of hopelessness, sadness and loss of interest in things they usually enjoy [2]. Similarly, anxiety can be described as a normal reaction to stress whilst anxiety disorders involve excessive fear or anxiety. Anxiety disorders are the most common of mental disorders [3]. The impact of these conditions on quality of life is significant. They are associated with decreased productivity, social isolation, and, in severe cases, suicide [4,5]. Among undergraduate health science students, the prevalence of depression and anxiety is exceptionally high, likely due to the demanding academic curriculum, clinical rotations, and personal stressors [6,7]. University students are often confronted with psychological challenges, which have emerged as a public health concern [6,8]. Published reports indicate increased mental health problems among university students, including symptoms of depression and anxiety [9,10,11]. Anxiety and depression are often comorbid, and the presence of one typically increases the risk of the other occurring over time [12]. A survey conducted in the United States reported that nearly half of college students met the criteria for a mental health condition in the past year [9]. Similarly, in 2011, a survey in Alberta, Canada, showed that about 800 students reported a feeling of hopelessness and anxiety, while 34.2% felt depressed during the 12 months prior to survey participation [8].

The prevalence of depression and anxiety reported among university students varies by over 20% [10,11,13]. A cross-sectional study reported that 45.3% of university students, including health science students in North America, had symptoms of depression, and 48.1% had anxiety [14]. In another study conducted in Asia, 66.86% of participants had a diagnosis of depression, and 57.39% had anxiety [15]. An African study conducted among medical students found that 88.8% of participants experienced depression, while 82.6% of participants experienced anxiety [6]. Moreover, higher estimates of anxiety (75.5%) [16] and depression (84.4%) were reported in a cross-sectional survey of medical students [17].

Most studies have focused on common psychiatric conditions, including anxiety and depression among medical students [18,19,20], and emphasize the magnitude of mental health challenges in this context. Published research indicates that health science students, including students studying medicine, pharmacy, nursing, physiotherapy, occupational therapy, and clinical psychology, experience a high level of depression and anxiety due to increased workload [21] and other factors such as the intensity of the course work and exams [21,22,23,24]. The literature also suggests that female health sciences students experience more depression and anxiety than their male counterparts [19]. In addition, a history of family mental illness, low family income, and stress levels affected depression and anxiety among medical students [25,26]. This scoping review aims to identify, document, and analyze the literature on the prevalence and determinants of anxiety and depression among health sciences students and identify gaps in knowledge for future research.

The focus on health sciences students was deemed valuable given their distinct risk profiles, the implications of poor mental health on their clinical practice, and the utility of this analysis in guiding supportive reforms in medical education. Existing research indicates health science students face exceptionally high rates of depression and anxiety due to demanding academics, clinical rotations, personal stressors, and other unique pressures [27]. The psychological wellbeing of future healthcare professionals is particularly crucial given its impacts on quality of care, empathy, and medical errors. Hence it is a significant public health concern. Understanding variables linked to adverse mental health outcomes in health sciences students can inform tailored interventions and policy changes in their academic training environments. While there is substantial literature on mental health issues among general university students [28], synthesized evidence specifically among health sciences students is lacking. The research question we aim to answer is “what are the prevalence and correlates of anxiety and depression among health science students in different countries?”

## 2. Methods

This scoping review followed the standards of Preferred Reporting Items for Systematic Reviews and Meta-Analyses (PRISMA) extension for scoping reviews [29] and Arksey and O’Malley’s five-stage approach to scoping reviews. Arksey and O’Malley’s five stages are researching questions, searching for relevant studies, study selection, charting the data, and collating, summarizing, and reporting the results [30]. A comprehensive search strategy ensured transparency, replicability, and reliability.

Scoping reviews are optimal for mapping research areas that have not yet been extensively reviewed or exhibit complex, heterogeneous evidence. This approach aligned well with the broad research question examining the prevalence and correlations across diverse geographic settings. Scoping reviews allow the inclusion of all study designs, rather than just clinical trials as in systematic reviews [31]. This enabled a comprehensive overview of the observational epidemiological research on this public health issue among students. Considering the methodological and population variations across studies, the scoping review methodology enabled maximum inclusivity of prevalent literature from disparate global contexts and was optimally suited to broadly map the research area and inform future systematic investigations.

### 2.1. Searching for Relevant Studies

Relevant terms were used to identify and choose articles in databases: MEDLINE (Medical Literature Analysis and Retrieval System Online; Ovid MEDLINE ALL), PubMed, EMBASE (Excerpta Medica Database; Ovid interface), CINAHL (Cumulative Index of Nursing and Allied Health Literature; EBSCOhost interface), and Scopus Elsevier. These databases were selected to cover the key literatures in medical, health, nursing, allied health and broader scientific fields. They represent leading databases indexing research output globally across these disciplines and student populations. The search was limited to English language original studies, peer-reviewed quantitative articles as they aligned with the study eligibility criteria. Keywords representing the concepts of depression and anxiety among university or college students and their correlates and prevalence were applied in the search. Appendix A shows the specific MeSH terms, keywords and descriptors included in the search. The database search was completed on 20 December 2022.

### 2.2. Study Selection

**Figure 1:**
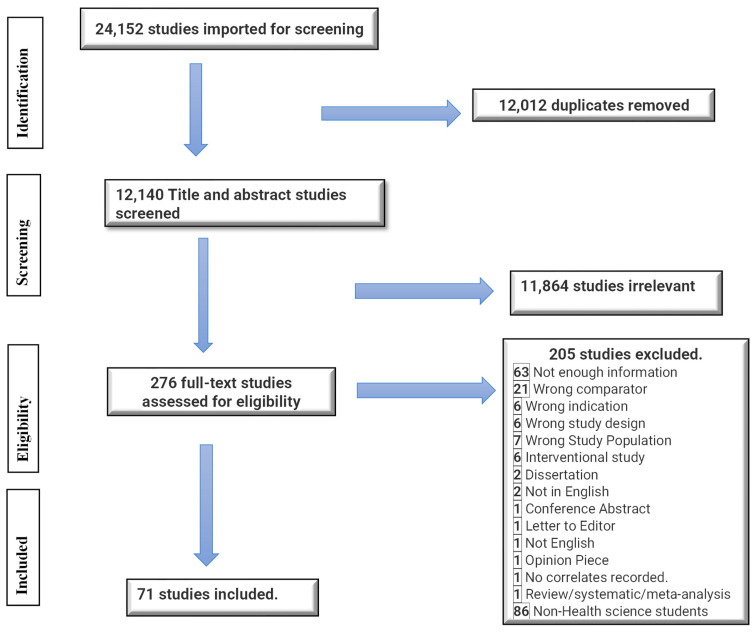
PRISMA flow chart.

Studies were deemed eligible for inclusion in this scoping review if they addressed the prevalence and correlates of depression or anxiety among health science students. The search was limited to English language original studies, peer-reviewed quantitative articles, and studies in which study participants were not tertiary, university, college, or medical students. Papers were also excluded if they were case reports, meta-analyses, systematic reviews, interventional studies or outcomes, commentaries, editorials, opinion pieces, or grey literature, including graduate student theses, non-peer-reviewed studies, non-research articles or conference reports or opinion pieces, commentaries, and editorials. The search was limited by publication year, from 2017 to 2022. This 6-year timeframe was selected to provide a contemporary overview of recent evidence while ensuring a sufficient volume of literature to map key prevalence and correlates. Two researchers independently reviewed citations during the title, abstract screening, and full-text review phases, and conflict was resolved through discussion and consensus. A total of 276 articles were initially identified for full-text review. After a thorough assessment, 205 articles were excluded, including 86 non health science students leaving a final selection of 71 articles for the comprehensive review. The PRISMA flow diagram summarizes this information in detail (Figure 1).

### 2.3. Data Charting and Extraction Process

Data were extracted for each article based on the following domains: author(s) name, year of publication, country of study, population/sample size [N], study design, assessment tools used, age, main findings, and conclusions.

### 2.4. Collating, Summarizing, and Reporting the Results

This scoping review gives an overview of existing evidence on the prevalence and the correlates of anxiety and depression among health sciences students. All relevant data were organized into tables and validated by at least two team members. The characteristics and results reported in each included article were summarized.

## 3. Results

### 3.1. Study Characteristics

**Figure 2:**
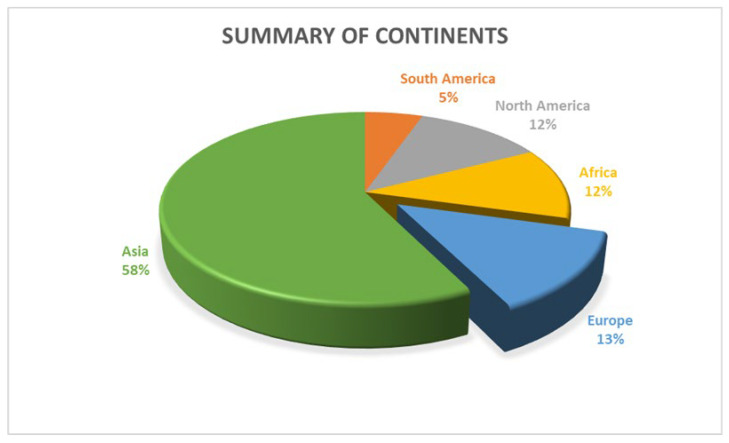
Summary of continents.

The search strategy identified 24,152 citations. The Covidence software [32] was used to automatically remove 12,012 duplicates. Two hundred and seventy-six studies remained for full-text screening, and 157 of these were eligible for inclusion. This paper focuses on the seventy-one studies on health sciences students. This included a total of 36,271 participants, who were all health science-related students. The sample size for the individual article from Table 1 and Table 2 ranged from 77 to 2798 participants, with an age range from 16 years to 54 years. The minimum response rate was 9.4%, and the maximum was 100%, with a median response rate of 76.4%. The articles included studies from 2017 to 2022. Most of the studies were conducted in Asia (58%), followed by Europe (13%), North America (12%), and Africa (12%). In contrast, South American studies represented 5%, as shown in Figure 2.

### 3.2. Number of Studies and Scales Used

Health science students reported in our study includes students in medicine, health sciences, pharmacy, dentistry, nursing, and allied health sciences, midwifery and health management, veterinary, physiotherapy, speech therapy, and occupational therapy, optometry, social work, dietetics and kinesiology. Overall, 43 reported on the prevalence and correlates of anxiety whilst 62 studies reported on the prevalence and correlates of depression. Thirty-one studies focused on both anxiety and depression prevalence and correlates. The Beck Anxiety Inventory Scale was used in eight studies; the Beck Depression Inventory Scale was used in 19 studies, whilst the Hospital Anxiety Scale was used in 10 studies. The Hospital Depression Scale was used in 10 studies, the Patient Health Questionnaire 9 (PHQ) was used in 17 studies, and the Depression, Anxiety, and Stress Scale was used in 13 studies. The Generalized Anxiety Disorder Scale was used in five studies, and the Center for Epidemiological Studies Depression (CESD-R) was used in three studies. The following scales were used in one study: Personality Inventory, Profile of Mood States Short Form, Visual Analog Scale, Positive Orientation Scale, Courtauld Emotional Scale, Subjective Happiness Scale, COVID-induced Anxiety Scale, and State-Trait Anxiety Inventory for Adults (STAI).

### 3.3. Prevalence and Correlates of Anxiety and Depression

Anxiety and depression are usually comorbid, and the presence of one normally increases the risk of the other occurring over time [12]. Table 1 and Table 2 indicate that the highest prevalence of anxiety was 82.6% [6], and the lowest prevalence was 5.8% [19], with a median of 44.25%. The highest prevalence of anxiety was reported among the Asian ethnic groups and the lowest among the Igbo ethnic group of West Africa [47]. The prevalence of depression ranged from a low of 2.1% for severe depressive symptoms [63] to a high of 88.8% depression [6], with a median for depression was 34.8%. In terms of gender, the median prevalence of anxiety among female health science students was 48.1%, which is slightly higher than the median found in the general health science student population of 44.25%. For studies from 2017 to 2019, the prevalence of depression ranged from 4.4% severe or extreme depression to 66% depression [34,48,57]. Similarly, the prevalence of anxiety reported in our study from 2017 to 2019 range from 5.8% moderate to severe anxiety to 74% anxiety [37,60]. Some correlates of depression for studies between 2017 and 2019 includes academic burnout, academic incompetency, female gender, breakups in relationships, perceived financial burden, poor academic performance or lower academic achievement, poor relationship with peers, the pressure of passing exams, fear of stepping into the real world of medicine, third-year student, moderate or poor socioeconomic status and being married [20,23,38,60,67,74,79,80]. The rates of depression were highest in year two and year three and lowest in year one and year four [57]. Burnout plays a significant role in the rate of depression experienced by health science students. For instance, an increase in burnout increased depression, when there is low burnout the prevalence of depression was 13%, for intermediate burnout the prevalence of depression was 38%,and for those in the high burnout category the prevalence of depression was 66% [48]. Correlates of anxiety for the 2017 to 2019 studies included poor relationship with peers, the pressure of passing exams, fear of stepping into the real world of medicine, low optimism, poor academic performance, female gender, year of study, studying all night before the exam, extensive course load, and low grade point average [54,60,67,70,84]. Satisfaction with faculty and peer relationships also affected both depression and anxiety prevalence among dental students [41]. History of major trauma or psychiatric events increased the possibility of depression among medical school participants [34].

Most of the studies in our scoping review were conducted from 2020 to 2022. The prevalence of depression was ranged from 2.12% of students with severe depressive symptoms or 5.5% with depression to 88.8% [6,63,77]. The prevalence of anxiety ranged from 11.8% to 82.6% [6,77]. The common correlates of anxiety from 2020 to 2022 included dissatisfaction with the course, being in the exam period, female gender, age, students in lower years of study, perceived poor academic performance, history of a mental problem and concurrent physical illness, possible COVID-19 exposure, depression, personal burnout, lower grade point average, experience of COVID-19 symptoms, teaching and learning-related stressors [15,24,39,68,72,77]. Similarly, the correlates of depression from the 2020 to 2022 studies included students in lower years of study, perceived poor academic performance, history of a mental problem and concurrent physical illness, possible COVID-19 exposure, family history of depression, female gender, older age, student academic year, high emotional exhaustion, high cynicism, burnout, age, financial crisis in family, preclinical years (first and second year) compared to third, fourth, and final year), anxiety, personal burnout, work-related burnout, lower grade point average, and experience of COVID-19 symptoms [15,18,33,36,39,46,68,72,77,81].

The correlates of anxiety and depression among health science students from Table 1 and Table 2 can be summarized into sociodemographic factors such as age, sex, gender, relationships, ethnicity and family history, personal health conditions, academic issues, and socioeconomic issues.

## 4. Discussion

### 4.1. Prevalence and Correlates of Anxiety and Depression among Health Science Students

Anxiety symptoms are common among health sciences students, with a significant proportion of undergraduate medical students being anxious [49,89]. Published research reports that about one in three medical students globally have anxiety, and this prevalence rate is reportedly higher than the general population [90]. The prevalence of anxiety among the health science students in this scoping review ranged from 5.8% to 82.6% [6,19], with a median of 44.25%.

The disproportionately higher volume of evidence from Asian countries compared to other regions reveals some key implications. It likely reflects underlying variability in mental health awareness and research prioritization across regions. Cultural differences in stigma and openness regarding psychological issues may also contribute to more recognized cases in some societies. Nevertheless, the underrepresentation of Western and African contexts is concerning given their significant student populations. It suggests the need to direct more attention toward understanding depression/anxiety prevalence and relationships in these settings through systematic studies with consistent methodologies. As medical education globalizes, a balanced geographic distribution of mental health research across student communities will be increasingly beneficial. Ultimately a nuanced, culturally aware analysis accounting for societal variances underpinning any geographic differences can help inform customized interventions to support student wellness.

The huge variance between prevalence reported in our scoping review may be attributed to the methodological differences between studies or differences in clinical scales used in the measurement of anxiety and the time the research was conducted [91,92]. It is also worth mentioning that the scoping review search strategy was limited by the year of publication which may contribute to the huge disparity in the prevalence. Our scoping review also reported a median of 44.25% which is higher than the global prevalence of anxiety reported in a meta-analysis 33.8% (95% Confidence Interval: 29.2–38.7%) [90] This shows that health science students may experience increased anxiety compared to other university students. The prevalence of depression ranged from a low of 2.1% for severe depressive symptoms [63] to a high depression rate of 88.8% [6], with a median for depression was 34.8%.

### 4.2. Sociodemographic Correlates of Anxiety and Depression among Health Science Students

In terms of gender, the median prevalence of anxiety among female health science students was 48.1%, which is slightly higher than the median found in the general health science student population of 44.25%. This suggests that anxiety among female health science students may be higher than their male counterparts. This is consistent with what was reported by one study, which showed that female medical students experience more anxiety than their male counterparts [19]. Another study among medical undergraduates [89] also reported that females have higher anxiety scores than their male counterparts. This is in line with other reviews [93] which suggest that females are twice as likely to develop an anxiety disorder compared to males. Other studies have also suggested that biological factors such as stress responsiveness significantly contribute to the gender differentiation in some expressions of both depression and anxiety with female being affected substantially [94]. The prevalence of anxiety symptoms among college students remained relatively high [95]. Some studies reported that the prevalence of depression is higher among female health science students compared to their male counterparts [6,80]. The higher prevalence of depression among female health sciences students is consistent with the prevalence of depression reported among the general public [96]. Furthermore, more generally, in the health sector, women have been reported to have a higher risk of experiencing both anxiety and depression compared to men [97]. With respect to age, one study among the public reported that older participants had a significantly lower anxiety score than their younger counterparts [98]. The study also suggested that age might moderate the effects of anxiety since maturity and experience associated with aging may cause a reduction in anxiety [98]. However, in this scoping review, a relatively lower prevalence of anxiety, 11.8% to 66.86% [15,50,77], was reported among health science students who were aged 20–25 years.

Among the female gender, relationships with peers in the year of study slightly reduced anxiety (51%) [67]. On the contrary, some studies have suggested that relationship status does not independently predict mental health issues [5,99]. Other general relationships, such as satisfaction with faculty and peer relationships, still led to a considerably high prevalence of anxiety (66.8%), with dissatisfaction with administration leading to even higher anxiety at 74% [60]. With respect to ethnicity, one study reported that anxiety is prevalent among medical students from the Middle East and Asia [90], with the highest prevalence reported among the Asian ethnic groups and the lowest anxiety prevalence (25%) reported among the Igbo ethnic group [47]. Another study reported that racial/ethnic minority health science students are generally less likely to report less anxiety than Whites [100]. As reported for anxiety, ethnic minority health science students are generally less likely to report depression relative to Whites, although there is variability in the prevalence of depression among ethnic minorities ranging from as low as 14.3% among the Igbo group 14.3% [47] to as high as 40.0% among the Arab group [67,82].

### 4.3. Personal and Health Conditions as Correlates of Anxiety and Depression

Physical health activities, including exercise, are usually associated with good general health. In one study, 63% of medical students who lack physical activities were classified as having high state anxiety and contributory factors such as living off-campus, sleeping five hours or less and smoking [71]. On the other hand, a slightly lower anxiety prevalence (41.7%) was reported in another study where students had reduced engagement in physical and other leisure activities, poor sleep quality, and poor self-perceived mental health [88]. Another study identified that a history of mental health problems, use of psychotropic drugs, stressors in schools, and perceived stress correlated with the presence of moderate to high anxiety among medical students [77]. Furthermore, another study identified that participants who reported less than seven hours of sleep per night, worse general health, higher stress, or perceived lack of control had higher rates of anxiety [51]. Other studies have reported that medical students who smoke [65,71] and those with high consumption of energy drinks [66] have higher rates of anxiety. These cohorts may be struggling with managing academic work or other unmanaged stressors leading to reduced sleep, contributing to increased anxiety. According to one study, sleep deprivation, whether total or not, significantly increased state anxiety levels [101]. With respect to the personal and health conditions which are predictors of depression, smoking is often linked to depression [102], and a higher prevalence of depression has been reported among health science students who smoke [6]. Other studies have also reported a higher prevalence of depression among students who smoke and use psychotropic medications [26], those who use alcohol [65], and those who have a history of psychiatric disorders or attempted suicide [7,77]. Furthermore, other studies reported higher depression prevalence in health science students with a history of mental health problems [66], a history of trauma [34], and living with personal chronic disease [42,81].

The onset of the COVID pandemic affected healthcare workers and the general public alike. During the initial peak phase of COVID-19, more than 60% of US medical students screened positive for pandemic-related anxiety [61]. The highest prevalence of both anxiety and depression among medical students was 82.6% and 88.8%, respectively, reported in our study was during the COVID-19 crisis [6].

### 4.4. Academic-Related Issues as Predictors of Anxiety and Depression

Teaching and learning-related stressors and high academic distress have been correlated with moderate to high anxiety [86,87]. Stressors related to high academic achievement are some of the most challenging problems experienced by health science students, resulting in high anxiety [55,67]. Several studies suggest that the pressure to succeed and pass exams during the time of assessment contributes to heightened anxiety (72% to 82.6% prevalence) in health science students, especially during the third year [6,38,49]. Extensive coursework coupled with studying all night before exams increases anxiety prevalence [54]. One study suggests that students in their final year may experience slightly less anxiety 40% [103], whilst another study suggests that those in first or second-year educational level experience increased anxiety 51.3% [59]. Finally, medical students’ burnout may lead to anxiety [72]. Depression prevalence can vary depending on the year of study or academic achievement [57]. For example, lower depression prevalence (15.1% to 29.2%) has been reported during the lower year of study or in preclinical students [19,80] compared to the prevalence of 53.0% in the senior academic years [66]. However, when students are in a lower year of study but have poor performance, depression prevalence increases significantly to about 44.6% [39]. Other studies have reported that students’ academic achievement and low academic grades increased depression to between 40% and 45.9% [55,67], with the highest prevalence of depression of 88.8% resulting from low achievement scores [6]. Related to these, academic burnout has been reported to contribute significantly to a higher prevalence of depression among health science students [79].

### 4.5. Limitations of the Scoping Review

This scoping review has several limitations. The search strategy was limited by the year of publication. We focused the search on only English language databases, which may lead to some older studies and some relevant studies in other languages that may not have been included. In addition, the search strategy may have been biased towards health and sciences databases, and there is the probability that searching other bibliographic databases may have resulted in additional relevant studies. Again, different studies included in this scoping review used different screening tools, which may have implications for the prevalence of anxiety and depression [92]. Finally, study characteristics, there are significance differences in terms of study characteristics like the range of response rates, differences in year of study, and potential overrepresentation of certain age groups which may affect the prevalences of depression and anxiety reported.

Notwithstanding these limitations, this scoping review gives a significant perspective on the prevalence and correlates of anxiety and depression among health science students.

## 5. Conclusions and Implications for Future Research

This scoping review has identified the prevalence and determinants of anxiety and depression among health science students. Health science students face many clinical requirements, pressure to pass exams, and family expectations [60]. The wide range of prevalence of anxiety and depression highlights the potential multifactorial predictors and impact of these problems among health science students. It also highlights the need for further research to understand better the prevalence and correlates of anxiety and depression among health science students and to identify the factors unique to certain populations which contribute to higher or lower prevalence of anxiety and depression. Health science training programs need to be designed to reduce the stressors which culminate into anxiety and depression among the students. University authorities, educationists and policy leaders should come together to review strategies for assessment, which removes the focus from examinations and high academic achievements and rather focus on professionalism, competence, good clinical judgement, and student wellbeing. Interventions and programs which provide cognitive behavioral therapy may also help reduce anxiety and depression and help improve mental health literacy and coping skills of health science students. Innovative interventions which are readily accessible and inexpensive may be developed, assessed, and offered to this cohort, especially those affected by anxiety and depression to improve their psychological well-being. Given that almost all health science students may be conversant with mobile technology, particularly texting and email messaging, these modes of healthcare delivery present a unique opportunity to offer support for their mental health [104]. One such program, ResilienceNHope, is an evidence-based text and email messaging innovation. It may be an appropriate mechanism for reaching and supporting college students to close the existing psychological treatment gap and improve their mental health literacy. In addition, several interventions have been reported to reduce psychological symptoms including mindfulness-based interventions alone or in combination with yoga, Cognitive Behavioral Therapy (CBT), and sport-based physical activity [105]. These can also be explored and adopted among health science students to improve their mental well-being. Considering the high prevalence of both anxiety and depression among the health science students, implementing such interventions will have potential long-term benefits for both the health science students and the broader healthcare field.

Health science students should be encouraged through advocacy groups to seek help when feeling stressed or overwhelmed to reduce the possibility of burnout or subsequent anxiety or depression. The huge disproportion in the number of studies published in different continents helps to appreciate that more research on depression and anxiety among health science students are conducted in Asia, but only 5% of research on this topic occurs in South America. This also beckon on researchers in these countries to refocus their attention on these cohorts. Considering the limitations of a scoping review, future studies may consider a systematic review with metanalysis.

## Figures and Tables

**Table 1:** Summary of studies reporting on the prevalence and correlates of anxiety and depression among health sciences students.

Authors/Year	Country	Study Design	Population Sample/Sample Size (Response Rate)	Students’ Age Range/Mean	Scales Used	Key Findings	
						Correlates of Anxiety/Depression	Prevalence of Anxiety/Depression
Alves et al., 2021 [24]	Brazil	Cross-sectional	Health 493 RR = 22.5%	Mean age of 23.1 years	The Beck Anxiety Inventory	Live with parents Dissatisfied with the course and being in the exam period	Severe anxiety 28.0%, Moderate anxiety 29.8%
Al-Maashani et al., 2020 [33]	Oman	Cross-sectional	197/1041 medical students RR = 18.9%	NR	Patient Health Questionnaire-9 (PHQ-9)	Female gender Family history of depression	The prevalence of depressive symptoms was 41.3%
Adhikari et al., 2021 [18]	Nepal	Cross-sectional	223 medical students	NR	Patient Health Questionnaire	Preclinical years (first and second year) compared to third, fourth and final year. Age less than 24	The prevalence of depression among medical students was 23.3%. (17.7–28.9 at 95% interval)
Albajjar et al., 2019 [34]	Saudi Arabia	Cross-sectional	161/306 medical students RR = 52.6%	AR = 19–26 Mean of 22.03 ± 1.94 years	Becks Depression Inventory	History of domestic abuse or violence History of major trauma or psychiatric event	The prevalence of depression was 53.8% Depression was mild in 25.8% and severe or extreme in 4.4% of the participants
AlShamlan et al., 2020 [35]	Saudi Arabia	Cross-sectional	527 medical students	NR	Patient Health Questionnaire (PHQ-9)	Female gender Students who perceived that they were not yet ready for their future specialities.	The prevalence of depression was found to be 39.27%
Alkhamees et al., 2020 [36]	Saudi Arabia	Cross-sectional	153 medical students	NR	Maslach Burnout Inventory Patient Health Questionnaire (PHQ-9)	Older Age Female Gender Student academic year High emotional exhaustion High cynicism Burnout	The current study assessed the prevalence of burnout and depression among medical students at UCM using the MBI and the PHQ-9. The estimated prevalence was 5.6% and 50.2%, respectively
Azad et al., 2017 [37]	Pakistan	Cross-sectional	150/415 medical students RR = 36.1%	AR = 17–26 Mean = 20.6 ± 0.88	Beck Anxiety Scale	Time of assessment Female gender	About 19% of the students had moderate to severe anxiety
Ahad et al., 2021 [15]	India	Cross-sectional	507 dental students RR = 65.3%	AR = 17–32 Mean age of 22.04 ± 2.20 years	Depression, Anxiety, and Stress Scale (DASS)-42	Depression: age is a strong positive predictor for anxiety and depression Anxiety: staying in the hostel was a positive predictor of anxiety	Anxiety was 66.86% (n = 339) Depression (57.39%)
Azim et al., 2019 [38]	Pakistan	Mixed-method qualitative-quantitative study	188/270 (70%) medical students	AR = 18–25 Mean = 21.4 ± 2.2 years	Depression, Anxiety, and Stress Scale-21	Third-year student Moderate or poor socioeconomic status	The prevalence of anxiety was 72%.
Abed et al., 2022 [6]	Egypt	Cross-sectional	597 medical students	NR	Depression, Anxiety, and Stress Scale (DASS-21)	Depression/Anxiety: Smoking Female gender In the third year Student average achievement score Social activity Transportation	Anxiety: Out of the included students, 82.6% experienced anxiety Depression: Out of the included students, 88.8% experienced depression
Aluh et al., 2020 [39]	Nigeria	cross-sectional	408 pharmacy students RR = 13.32%	Mean age = 22.57 ± 3.39	Depression, Anxiety and Stress Scale (DASS)	Anxiety/depression: Students in lower years of study perceived poor academic performance	The overall prevalence of depression, anxiety and stress was 44.6%, 63.5%, and 35%, respectively
Adhikari et al., 2017 [19]	Nepal	Cross-sectional	343 medical students	AR = 18–25	Hospital Anxiety Scale Patient Health Questionnaire	Anxiety: Female Gender Preclinical students Depression: Preclinical years (first and second year) compared to third, fourth and final year) Age less than 24	Anxiety: There was a 5.8% prevalence rate for anxiety. Depression: The prevalence of depression among medical students was 23.3%. (17.7–28.9 at 95% interval)
Bertani et al., 2020 [40]	Italy	Cross-sectional	459/944 (48%) medical students	AR = 19–50 Mean = 23 plus or minus 3	Hospital Anxiety and Depression Scale (HADS) Personality Inventory for DSM-5	Anxiety/depression: Personality traits Namely detachment negative effect Cognitive enhancers	The prevalence of anxiety was 20% The prevalence of depression was 7%
Basheti et al., 2021 [26]	Jordan	Cross-sectional	450 healthcare students (medicine, dentistry, Pharm.D., pharmacy, nursing, and other)	Mean = 21.62	Hospital Anxiety and Depression Scale (HADS)	Smoking Lower family income Use of medications	22.4% of students were classified as having anxiety 33.8% of students were classified to have depression
Basudan et al., 2017 [41]	Saudi Arabia	Cross-sectional	247 dental students		Anxiety scale (DASS-21) Depression scale (DASS-21)	Anxiety/Depression: Male Gender-Low anxiety Satisfaction with faculty relationships Satisfaction with peer relationships Dentistry is the first choice for the field of study	Prevalence of anxiety was 66.8%. The prevalence of depression was 55.9%.
Bert et al., (2020) [42]	Italy	Multicenter cross-sectional	2396 medical students RR = 95%	Median age was 22	Beck Depression Inventory-II (BDI-II)	Age being female bisexual/asexual orientation, living with partner/housemates poor economic status less than 90 min of weekly exercise relatives with psychiatric disorders personal chronic disease judging medical school choice negatively unsatisfying friendships with classmates competitive and hostile climate among classmates thinking that medical school hinders specific activities and being worried about not measuring up to the profession	Depression—29.5%
Bresolin et al., 2020 [22]	Brazil	Cross-sectional	792 healthcare students (Nursing, Pharmacy, Physiotherapy, Speech Therapy, Medicine, Dentistry and Occupational Therapy)	NR	Beck Depression Inventory	Non-performance of physical and leisure activities Speech therapy Nursing courses	Depression was moderate to severe in 23.6% of students
Boolani et al., 2021 [17]	United States	Cross-sectional	77 health students	AR = 18–45 Mean = 25.83	30-item Profile of Mood States Short Form (POMS-SF)	Worse sleep quality Increased sitting time Trait physical fatigue	65/77 (84.4%) of participants reported feelings of depression
Biswas et al., 2022 [43]	Bangladesh	Cross-sectional	425 medical students RR- = 93.2%.	Age = 22 years	Patient Health Questionnaire-9	Female students Those who struggled to stay away from social media Those who tried to be optimistic to maintain better psychology those who always had sleeping difficulty in the last four weeks	Prevalence of depression was 80.2%
Coelho LDS et al., 2021 [44]	Brazil	Cross-sectional	192	Mean age = 21.44 (±3.56)	Beck Depression and Anxiety Inventories	Anxiety/depression: Female gender Student from 6th–10th semester Psychotropic drug use	On the anxiety scale, a minimal classification was predominant (30.21%) On the depression scale, severe symptoms were the most frequent (30.73%)
Çelik et al., 2019 [45]	Turkey	Cross-sectional	445 health students (nursing, midwifery and health management)	Mean age = 21.0 ± 2.0	Beck Depression Inventory	Sleep quality: As sleep quality deteriorates, the level of depression also increases Academic failure Level of income Smokers or alcohol drinkers	The risk of depressive symptoms in students with poor sleep quality was 3.28 times.
Elsawy et al., 2020 [46]	Egypt	Cross-sectional	390 medical students	NR	Beck Depression Inventory Scale—2 (BDI-2)	Female gender The presence of mental illness Not having someone to talk to when under stress Experiencing stressful life event(s) during the previous six months Not being satisfied with the socioeconomic level Reporting that the surrounding environment is not suitable for studying Not specifying a grade to achieve Extreme dissatisfaction with students results	The prevalence of moderate and severe depression was 27.9% and 17.2, respectively The prevalence of depression was high among medical students
Falade et al., 2020 [47]	Nigeria	Cross-sectional	944 medical students RR = 97.8%	16 and 32 years, the overall mean was 21 ± 3.0 years	Hospital Anxiety and Depression Scale	Being a student receiving less than one dollar equivalent per day as an allowance A student from the Igbo ethnic group	Prevalence of anxiety was 25% Depression—14.3%
Fitzpatrick et al., 2019 [48]	Ireland	Cross-sectional	269 medical students	NR	Beck Depression Inventory—Fast Screen	Increase burnout increases depression	There was a 39% prevalence of depression caseness Low burnout had 13% depression Intermediate burnout had 38% depression The high burnout category had 66% depression.
Fawzy et al. (2017) [49]	Egypt	Cross-sectional	700 medical students RR = 100%	Mean age of 21.22 ± 1.632 years	Depression, Anxiety, and Stress Scale-21 (DASS-21)	Anxiety/Depression: Females Gender Those living in the University campus/students’ residence facility In the preclinical years lower academic achievement had higher scores in DASS	Prevalence of anxiety was 73% Prevalence of depression was 65%
Gupta et al., 2021 [14]	United States	Cross-sectional	438 (33.4%) medical students	NR	PHQ-9 and GAD-7	Anxiety/depression: Medical students Female gender	The prevalence of anxiety was 48.1%. Moderate to severe anxiety was 20.3% The prevalence of depression was 45.3%. Moderate to severe depression was 17.2%
Gan et al., 2019 [50]	Malaysia	Cross-sectional	149 medical students RR = 96.7%	AR = 22–24	Hospital Anxiety and Depression Scale (HADS)	Anxiety and depression were associated with significantly poorer QOL. Students with depression symptoms were associated with lower physical, psychological and environmental domain scores, whereas those with anxiety had lower psychological, social and environmental scores.	The prevalence rates of anxiety and depression were 33% and 11%, respectively
Hoying et al., 2020 [51]	United States	Cross-sectional	197 Health sciences students (Dentistry, Medicine, Nursing, Optometry, Pharmacy, Social Work, and Veterinary Medicine)	Mean age = 24.5 years	General Anxiety Disorder Scale Patient Health Questionnaire-9	Less than seven hr. of sleep per night Worse general health Lower healthy lifestyle beliefs Lower healthy lifestyle behaviours Higher stress Perceived lack of control	Anxiety—14% Depression—17%
Hanoon et al., 2021 [52]	Baghdad	Cross-sectional	301 medical students		Center for Epidemiological Studies Depression Scale (CESD-R)	Stage of the study Female gender Social relationship	The overall prevalence of depressive symptoms is 55.48%.
Junaid et al., 2020 [53]	Saudi Arabia	cross-sectional	247 medical students RR-90%	NR	Beck Anxiety Inventory (BAI)	Females Low academic grades Those in the final year	Anxiety—40%
Khoshhal et al., 2017 [54]	Saudi Arabia	Cross-sectional	111 Medical students RR = 89%	NR	Visual Analog Scale (VAS)	Studying all night before the exam Extensive course load Female gender	65% of students experienced exam anxiety
Kathem et al., 2021 [55]	Iraq	Cross-sectional	750 healthcare students (Pharmacy and medicine)	AR = 19–23	Hospital Anxiety Scale Hospital Depression Scale	Lower sleep hours at night Academic achievement Colleagues and family social support during exams	More than one-half (52.1%) of the participants had scores that indicated anxiety symptoms In comparison, 20.1% had scores that indicated borderline anxiety symptoms Approximately forty-six per cent (45.9%) of the participants had scores that indicated depression symptoms, and one-quarter (24.8%) had scores that indicated depression borderline symptoms
Kumar et al., 2017 [56]	India	Cross-sectional	444 medical students RR = 88.8%	NR	Beck Depression Inventory Scale	Severe stress level Those without interpersonal problems Low levels of perceived interpersonal support Age, study year, satisfaction with the major, parental relationship, and mother’s education among medical student Smoking, alcohol use, family history of depression, academic achievement, and interpersonal problems in the family	Depression—48.4%
Killinger et al., 2017 [57]	North America	Cross-sectional	1245 veterinary medical students	AR = 18–54	Centre for Epidemiological Studies Depression Scale (CES-D)	Female gender Year in the program—Rates of depression were highest in year two and year three and lowest in year one and year 4	66% of the population had symptoms ofdepression
Kupcewicz et al., 2022 [58]	Poland Spain Slovakia	Cross-sectional	756 nursing students	Mean age = 21.20 years	Positive Orientation Scale Courtauld Emotional Scale	Age-Anxiety control increases with age. More time working on computer Family crisis	Depression was diagnosed in 35.8% of study participants
Kebede et al., 2019 [59]	Ethiopia	Cross-sectional	273 medical students	AR = 18–21	Hospital Anxiety and Depression Scale	Female gender First-year educational level Second-year educational level Poor/low social support	The prevalence of anxiety and depression was 51.30%
Kumar et al. (2019) [60]	Karachi	Observational study	312 Medical students RR = 69.3%	Mean age = 22.74 ± 1.52 years	Depression Anxiety Stress Scale-21 (DASS-21)	The pressure of passing exams The pressure of living up to family’s expectations Fear of stepping into the real world of medicine Dissatisfaction with the administration	Anxiety—74% Depression—57.6%
Lee et al., 2021 [61]	United States	Cross-sectional	741 medical students	NR	General Anxiety Disorder Scale (GAD-7)	Female gender Being Asian	During the initial peak phase of COVID-19, over 60% of US medical students screened positive for pandemic-related anxiety
Lopez et al., 2017 [62]	Chile	Cross-sectional	235 health students (medical, nursing, and kinesiology)	AR = 18–34 Mean = 20.7 ± 3.41	Depression, Anxiety, and Stress Scale (DASS-21)	Personality organization dimensions	The prevalence of anxiety was 39% The prevalence of depression was 23%
Lu et al., (2022) [63]	China	Cross-sectional	519 Medical students RR = 50.39%	Mean = 22.76 ± 3.60	Patient Health Quesionnaire-9	Negative coping positive correlation with depression Perceived social support negative correlation with depression.	9.83%, 3.08%, and 2.12% of students had mild, moderate and severe depressive symptoms, respectively

RR—Response rate; BDI—Beck Depression Inventory; WHO—World Health Organization; AR—Age range; NR—Not recorded.

**Table 2:** Continuation of summary of studies reporting on the prevalence and correlates of anxiety and depression among health sciences students.

Authors/Year	Country	Study Design	Population Sample/Sample Size (Response Rate)	Students’ Age Range/Mean	Scales Used	Key Findings	
						Correlates of Anxiety/Depression	Prevalence of Anxiety/Depression
Milić et al., 2019 [64]	Croatia	Cross-sectional	562 medical and nursing students	AR = 20–24 Mean = 22	Patient health questionnaire (PHQ-9) General Anxiety Disorder Scale (GAD-7) Subjective Happiness Scale (SHS)	Emotional stability negatively correlated with anxiety and depression.	The prevalence of anxiety was 54.5% among Croatian medical students The prevalence of depression was 60.2% among Croatian medical students
Melaku et al., (2021) [65]	Ethiopia	cross-sectional	265 medical students RR = 98%	Mean = 22.03	Depression, Anxiety, Stress Scale-21 (DASS-21)	Anxiety: Gender-Males less likely to be depressed. Marital status-less likely to be depressed. Cigarette smoking-Increases depression. Depression: Low Monthly income-More depressed. Residency- Non-dormitory living respondents more likely to be depressed. Alcohol drinking-More likely to be depressed.	Anxiety—60.8% Depression—52.3%
Mirza et al., 2021 [66]	Saudi Arabia	Cross-sectional	231 medical students	Mean = 21.67 ± 1.56	Depression, anxiety and stress scale-21 items (DASS-21)	Anxiety: Female gender High consumption of energy drinks Spending more time on leisure activities and hobbies Long travel time from home to university Family conflicts Depression: Female gender History of psychiatric disorder Senior academic year Travel time from home to university Family conflicts at home	Approximately 54%, 53%, and 38% of participants were found to be suffering from anxiety, depression and stress.
Mahroon et al., 2018 [67]	Bahrain	cross-sectional	Medical 350 RR-87.6%	AR = 18 to 25	The Beck Anxiety Inventory The Beck Depression Inventory	Anxiety: Female gender Year of Study Academic performance Depression: Arab ethnicity Female gender relationship with peers Year of Study Academic performance	Prevalence of anxiety is 51% Prevalence of depression is 40%
Nakhostin-Ansari et al., 2020 [68]	Iran	Cross-sectional	323/500 (64.6%) medical students	Mean = 23.73.	Beck Anxiety Inventory (BAI) Beck Depression Inventory (BDI)	Female gender Lower grade point average Experience of COVID-19 symptoms.	The prevalence of anxiety was 38.1% The prevalence of depression was 27.6%
Nahas et al., 2019 [69]	Malaysia	Cross-sectional	365/425 (85.9%) health students (Medicine, Pharmacy, Dentistry, Nursing, and Allied Health Sciences students)	NR	Patient Health Questionnaire (PHQ-9)	Student’s origin- urban areas more likely to report depression.	The prevalence of depression was 36.4%
Nezam et al., 2020 [21]	Patna	Cross-sectional	2798/3100 (90.25%) medical, dental and students)	NR	Beck’s Depression Inventory 2	Dental students-higher prevalence than medical students.	The overall prevalence of depressive symptoms was found to be 47.78% Of the three streams, students belonging to the engineering stream (40.28%) showed a maximum prevalence of depressive symptoms, followed by dental (38.50%) and medical students (34.74%)
Nahar et al., 2019 [70]	Southeastern United States	Cross-sectional	264 veterinary students	Mean = 25.3 ± 3.21 years	Patient health questionnaire (PHQ-4)	Female Gender Grade point: Low Average Non-white	The prevalence of anxiety was 52.3% among veterinary students The prevalence of depression was 22.6% among veterinary students
Nebhinani et al., 2021 [16]	Western India	Cross-sectional survey	229 nursing students	AR = 21–25 Mean = 21.6 ± 2.8	COVID-induced anxiety scale Likert scale	Age- Positive association with the age of the students only (*p* = 0.001) Negative linear correlation between anxiety score and protective behavior score.	The prevalence of anxiety was 75.5%
Otim et al., 2021 [71]	United Arab Emirates	Cross-sectional	219 clinical training students	NR	State-Trait Anxiety Inventory for Adults (STAI)	Female gender Students living off-campus Students who slept for 5 h or less Students that reported no physical activity Current smoker	63% of the sample had high state anxiety, and 62% had high trait anxiety
Pokhrel et al., (2020) [72]	Nepal	cross-sectional	651 Medical students	Mean = 25 years	Nepali version of the Hospital Anxiety and Depression Scale	Anxiety: Female gender Depression Personal burnout Teaching and learning-related stressors History of mental illness Depression: Anxiety Personal burnout Work-related burnout	Anxiety—45.3% Depression—31%
Pukas et al., (2022) [73]	Germany	Cross-sectional	1103 medical students RR = 90.2%	Mean = 23.1	BDI-II	Neuroticism Insufficient emotional support Eating irregular meals Use of medication or drugs to calm down mental overload	Prevalence of depressive symptoms was 11% for mild, 5.6% for moderate and 2.4% for severe symptoms
Pham et al., 2019 [74]	Vietnam	cross-sectional	494 medical students RR = 78.8%	AR = 21 and above	Patient Health Questionnaire 9 (PHQ-9)	non-self-determined motivation perceived financial burden vigorous level of physical activity	The prevalence of self-reported depression was 15.2%
Patelarou et al., 2021 [75]	Greece, Spain, and Albania	Cross-sectional	787 nursing students	Mean = 22.7	Patient Health Questionnaire 9 (PHQ-9)	Decreased age Identifying as Spanish Living with people in high-risk groups Working status during the pandemic	1/3 of the nursing student population experienced depression
Patten 2021 [76]	US	Cross-sectional	611 Dietetics students	NR	Depression, Anxiety, and Stress Scale (DASS-21)	Significant sources of stress were postgraduation plans (including internships), managing time, dietetics courses, finances, and self-imposed expectations.	Depression (30%), anxiety (40%), and stress (27%)
Raghunathan et al., 2019 [23]	India	Cross-sectional	364 dental students	NR	Patient Health Questionnaire-9 (PHQ-9)	Being married Having a low and average level of course satisfaction Having close friends Female gender Breakups in relationships	The prevalence of depression was estimated at 26.9%.
Risal et al., 2020 [77]	Nepal	Cross-sectional	416 medical students	Mean age = 22.2	Hospital Anxiety and Depression Scale	History of a mental problem Concurrent physical illness possible COVID-19 exposure	The prevalence of anxiety was 11.8% The prevalence of depression was 5.5%
Santangelo et al., 2022 [78]	Italy	Cross-sectional	Nursing students 525	Mean age = 21.8	Quick Inventory of Depressive Symptomatology Self-Report Questionnaire (QIDS-SR16)	Female gender Low perceived economic status Low perceived health status To be a smoker	Per the scores obtained from the QIDSSR16 test, we can say that just over half of the sample (51.3%) does not exhibit depressive symptomatology
Shawahna et al., 2020 [7]	Palestine	Cross-sectional	286 medical students RR = 67.3%	Median age was 20, with an IQR of 3 years	Beck Anxiety Inventory (BAI) Beck Depression Inventory-II (BDI-II)	Anxiety: academic stage academic year 4 to 6 low BDI scores. mental health status Depression: Higher Grade Point Average–Low BDI score Low mental health status, Attempted suicide, Low religious commitment- higher BDI-II scores	21.3% had severe anxiety 9.1% had severe depression
Shrestha et al., (2019) [20]	Nepal	cross-sectional	217 medical students	NR = 18–29 years.	Beck’s Depression Index II	Academically incompetent Medical students progressed to their clinical years	Depression—27.2%
Silva et al., 2017 [79]	Portugal	Longitudinal study	2234/238 (74%) medical students		Beck Depression Inventory (BDI)	Academic burnout Anxiety traits Medicine choice Relationship patterns	The prevalence of depression ranged between 12.7% to 21.5%.
Suraj et al., 2021 [80]	Nigeria	Cross-sectional	279/285 (98%) medical students	AR = 21.75 ± 3.25 years Mean = 21.75	3-item Oslo Social Support Rating Scale	Female gender Age < 22 years Those at the lower level of study Poor social support Family history of depression History of depression	The prevalence of depression among medical students was 15.1%
Solanki et al., 2021 [81]	North India	Cross-sectional	414/500 (82.8%) medical students	AR = 17–29 Mean = 20.9	Centre for Epidemiologic Studies Depression Scale (CES-D) WHO Quality of Life—BREF questionnaire Smartphone Addiction Scale	Smartphone addiction Living with chronic disease They feel like their siblings or friends are more accomplished than themselves. Hospitalization of any family member for over 24 h in the last year Divorce or separation of parents Parental pressure for academic excellence Financial crisis in the family Romantic breakup	The prevalence of depression was 36.7%.
Tayefi et al., 2020 [82]	Iran	Cross-sectional	560 health students (medical and other health sciences)	Mean = 21.1 ± 5.3 years	Beck Depression Inventory Beck Anxiety Inventory General Health Questionnaire WHO wellbeing index	Ethnicity- Kurdish students more likely to have mild to severe depression symptoms compared to Persian students. Birthplace- Being born in the capital city lower anxiety. Maternal education level-Higher maternal education less likely anxiety. General psychiatric morbidity	About 29% (n = 161) of the students had mild to severe anxiety symptoms (20.7% mild, 7.1% moderate, and 0.9% severe) Overall, out of 560 health sciences students, 56 (10%) students had mild to severe depressive symptoms; of those, 37 (6.6%) were mild, 10 (1.8%) were moderate, and 9 (1.6%) were severe
Turan et al., 2021 [83]	Turkey	Cross-sectional	Nursing students 456	Mean = 21.09 ± 2.41	Anxiety scale Attitude Scale for Nursing Profession	Female gender, Third-year students, People who chose the profession willingly, Students who did not think about changing the profession of nursing and those who did not have anxieties about the profession	Anxiety was prevalent in 43.2% of the participants
van Venrooij et al., (2017) [84]	Netherlands	Cross-sectional	433 medical students RR = 33.0%	Mean = 21.2 years	Depression and anxiety-related symptoms and vitality using the Symptom Questionnaire-48 (SQ-48)	Low optimism Low happiness High need for recovery	Depression and anxiety related symptoms—46.0% Depression—27.0% Anxiety—29.1%
Van der Walt et al., 2020 [85]	South Africa	cross-sectional	473 medical students RR = 97.3%	Age = 22 years	Patient Health Questionnaire-9 Hospital Anxiety and Depression Scale	Students who undertook the 2017 mini-semester Female sex	36.4% were above the cut-off for major depressive disorder and 45.9% for anxiety disorder Reported rates of disorders diagnosed by a health professional were 25.0% for depressive disorder and 20.5% for anxiety disorder
Yusof et al., 2020 [25]	Malaysia	Cross-sectional	610 Pharmacy students RR = 20.3%	AR = 18–29 years	Depression Anxiety Stress Scale-42 (DASS-42)	Students who did not have depression, students who smoke, have separated parents, with a family history of mental illness, had a recent loss of someone close, and with lower GPAs were statistically significantly associated with depression	47.4% were having depression of different severity
Yuan et al., 2021 [86]	China	Cross-sectional	519/5550 medical students RR = 9.4%	AR = 16–42	Generalized Anxiety Disorder-7 (GAD-7) Patient Health Questionnaire-9 (PHQ-9) Simplified Coping Style Questionnaire (SCSQ) Perceived Stress Scale (PSS-10) Multidimensional Scale of Perceived Social Support (MSPSS) Revised Life Orientation Test (LOT-R) Resilience Scale- 14 (RS-14)	Stressors in school- Increase anxiety and depression. Negative coping style perceived stress	The prevalence of anxiety symptoms in the sample population was 28.5% The prevalence of depressive symptoms in the sample population was 31.6%
Zakeri et al., (2021) [87]	United States	Cross-sectional	238 pharmacy students RR = 63%	NR	Counselling Center Assessment of Psychological Symptoms instrument (CCAPS-62)	High academic distress and high family distress were associated with a greater probability of a student having high general anxiety.	Anxiety—50%
Zeng et al., 2019 [88]	China	Cross-sectional	544 nursing students RR = 89.9%	AR = 17–24 years	Depression, Anxiety and Stress Scale 21 (DASS 21)	Reduced engagement in physical and other leisure activities Poor sleep quality, experience of negative life events, poor self-perceived mental health	Depression—28.7% Anxiety—41.7%

## Data Availability

Not applicable.

## Appendix A

The specific MeSH terms, keyword and descriptors included in the search.: (depress* OR depression OR “depressive disorder” OR “depressive symptoms” OR “major depressive disorder”) OR (anxiety OR “anxiety disorder” OR “generalized anxiety disorder”), (predictors OR risk factors OR correlates OR predisposition OR determinants OR cause), AND (prevalence OR incidence OR epidemiology OR frequency OR occurrence OR statistics)AND (university student* OR undergraduate student* OR tertiary students” OR “higher education students” OR “college students” OR “post-secondary students”).

depress*.mp.KL	
limit 1 to yr	2017–2022
anxiety*.mp.	
limit 3 to yr	2017–2022
2 or 4	
college student*.mp.	
limit 6 to yr	2017–2022
university student*.mp.	
limit 8 to yr	2017–2022
undergraduate student*.mp.	
limit 10 to yr	2017–2022
post-secondary student*.mp.	
limit 12 to yr	2017–2022
tertiary student*.mp.	
limit 14 to yr	2017–2022
7 or 9 or 11 or 13 or 15	
prevalence*.mp.	
limit 17 to yr	2017–2022
correlate*.mp.	
limit 19 to yr	2017–2022
determinant*.mp.	
limit 21 to yr	2017–2022
relat*.mp.	
limit 23 to yr	2017–2022
incidence*.mp.	
limit 25 to yr	2017–2022
18 or 20 or 22 or 24 or 26	
5 and 16 and 2	
